# Five Years of Accurate PM_2.5_ Measurements Demonstrate the Value of Low-Cost PurpleAir Monitors in Areas Affected by Woodsmoke

**DOI:** 10.3390/ijerph20237127

**Published:** 2023-11-30

**Authors:** Dorothy L. Robinson, Nigel Goodman, Sotiris Vardoulakis

**Affiliations:** 1 Healthy Environments and Lives (HEAL) National Research Network, Canberra, ACT 2601, Australia; nigel.goodman@anu.edu.au (N.G.); sotiris.vardoulakis@anu.edu.au (S.V.); 2 College of Health and Medicine, The Australian National University, Canberra, ACT 2601, Australia

**Keywords:** woodsmoke, PM_2.5_, air pollution, spatial variation, low-cost pollution monitors, population exposure

## Abstract

Low-cost optical sensors are used in many countries to monitor fine particulate (PM_2.5_) air pollution, especially in cities and towns with large spatial and temporal variation due to woodsmoke pollution. Previous peer-reviewed research derived calibration equations for PurpleAir (PA) sensors by co-locating PA units at a government regulatory air pollution monitoring site in Armidale, NSW, Australia, a town where woodsmoke is the main source of PM_2.5_ pollution. The calibrations enabled the PA sensors to provide accurate estimates of PM_2.5_ that were almost identical to those from the NSW Government reference equipment and allowed the high levels of wintertime PM_2.5_ pollution and the substantial spatial and temporal variation from wood heaters to be quantified, as well as the estimated costs of premature mortality exceeding $10,000 per wood heater per year. This follow-up study evaluates eight PA sensors co-located at the same government site to check their accuracy over the following four years, using either the original calibrations, the default woodsmoke equation on the PA website for uncalibrated sensors, or the ALT-34 conversion equation (see text). Minimal calibration drift was observed, with year-round correlations, r = 0.98 ± 0.01, and root mean square error (RMSE) = 2.0 μg/m^3^ for daily average PA PM_2.5_ vs. reference equipment. The utitilty of the PA sensors without prior calibration at locations affected by woodsmoke was also demonstrated by the year-round correlations of 0.94 and low RMSE between PA (woodsmoke and ALT-34 conversions) and reference PM_2.5_ at the NSW Government monitoring sites in Orange and Gunnedah. To ensure the reliability of the PA data, basic quality checks are recommended, including the agreement of the two laser sensors in each PA unit and removing any transient spikes affecting only one sensor. In Armidale, from 2019 to 2022, the continuing high spatial variation in the PM_2.5_ levels observed during the colder months was many times higher than any discrepancies between the PA and reference measurements. Particularly unhealthy PM_2.5_ levels were noted in southern and eastern central Armidale. The measurements inside two older weatherboard houses in Armidale showed that high outdoor pollution resulted in high pollution inside the houses within 1–2 h. Daily average PM_2.5_ concentrations available on the PA website allow air pollution at different sites across regions (and countries) to be compared. Such comparisons revealed major elevations in PA PM_2.5_ at Gunnedah, Orange, Monash (Australian Capital Territory), and Christchurch (New Zealand) during the wood heating season. The data for Gunnedah and Muswellbrook suggest a slight underestimation of PM_2.5_ at other times of the year when there are proportionately more dust and other larger particles. A network of appropriately calibrated PA sensors can provide valuable information on the spatial and temporal variation in the air pollution that can be used to identify pollution hotspots, improve estimates of population exposure and health costs, and inform public policy.

## 1. Introduction

Many countries have air quality standards. In Australia, the desired environmental outcome was “ambient air quality that allows for the adequate protection of human health and well-being” [1]. This, and the desired environmental outcome of the follow-up legislation of “ambient air quality that minimises the risk of adverse health impacts from exposure to air pollution” [2], cannot be achieved unless air pollution is monitored both in areas that are considered representative of the population exposure as a whole, and in ‘hotspot’ areas, where pollution is likely to be unhealthy.

PM_2.5_ is often considered the most hazardous air pollutant. For example, the Lancet 2019 ‘Global Burden of Disease’ study attributed 4.51 million premature deaths worldwide to ambient air pollution; 92% were due to exposure to PM_2.5_ and 8% due to exposure to ozone [3]. The European Environment Agency’s ‘Health impacts of air pollution in Europe, 2022’ study attributed 238,000 premature deaths to PM_2.5_ exposure above the 2021 World Health Organization (WHO) annual guideline (5 μg/m^3^), compared to 49,000 premature deaths from nitrogen dioxide exposure above the WHO annual guideline (10 μg/m^3^) and 24,000 from acute ozone exposure [4].

Unhealthy PM_2.5_ air pollution levels with large spatial and temporal variation has been recorded in towns and cities affected by wood heater pollution in the USA [5], Canada [6], New Zealand [7], and Australia [8,9]. In Armidale, a regional town in New South Wales (NSW) with an urban centre population of approximately 21,300 [10], average PM_2.5_ pollution on 14 winter nights in 1996 increased from about 35 to 90 μg/m^3^ within 41 metres of driving from an undeveloped creek land to an area with houses beside the road [9]. Appropriately calibrated low-cost monitors can therefore provide valuable information on temporal and spatial variation in PM_2.5_ pollution, identify pollution hotspots (allowing remedial action to be considered at local scale), and contribute to improved population-wide exposure estimates.

Several studies, including two by the US Environmental Protection Agency (US EPA) have demonstrated the utility of PurpleAir (PA) [11,12,13,14,15] and other [16] low-cost sensors to accurately monitor PM_2.5_, when calibrated with data from reference monitors. Some governments have supported the research, deployment, and use of data from low-cost sensors, but others are operated by citizen scientists with limited opportunities for testing and calibration, including a large proportion of the 30,000 + PA units (current price USD 229) installed worldwide as of September 2021 [17].

For sensors that are not calibrated by co-location with reference equipment, the PA website (map.purpleair.com, accessed on 13 November 2023) calculates ‘conversions’ for specific aerosols, including ‘woodsmoke’, based on the average intercept and slope used to convert PA measurements to reference PM_2.5_ in the Armidale study, and the ALT conversion which converts particle counts recorded by the Plantower laser counters in different size categories to particle mass [12]. Although small differences in sensitivity were noted when the PA sensors were calibrated in Armidale, the differences were relatively minor, consistent with the fact that PA pre-tests laser counters before sensors are sold [18].

Because of the high spatial and temporal variation in wood heater pollution, a single reference PM_2.5_ monitor cannot provide an accurate assessment of the population exposure to PM_2.5_ from wood heaters. However, if the accuracy and reliability of PA sensors is established, they could provide a valuable resource to identify and evaluate spatial and temporal variation in air quality and help quantify population exposure for locations that are subject to high PM_2.5_ pollution from wood heaters.

**Figure A1:**
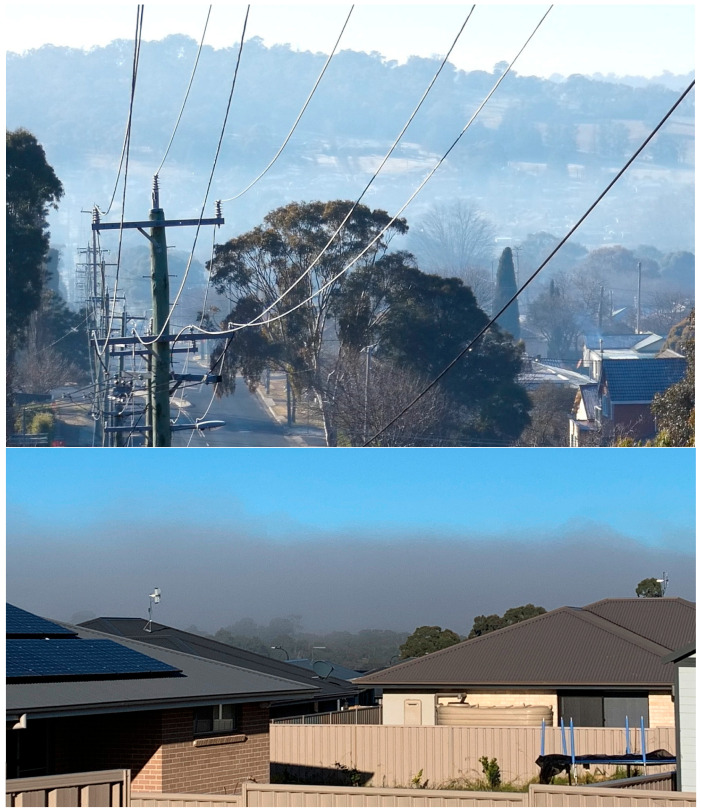
Armidale woodsmoke pollution, August 2021 (**top**), May 2020 (**bottom**).

The accurate characterization of population exposure for all locations affected by wood heater pollution is important because published research shows estimated health costs, including premature mortality, exceed AUD 10,000 per wood heater per year in Armidale [19] (see Figure A1 for examples of the pollution) and Greater Sydney [20], over AUD 4000 in Tasmania [21] and NZD 7000 in New Zealand (NZ) [22].

The present study evaluates the performance of seven PA sensors over four years after the initial Armidale calibration study using 2018 data [11,23], and four PA sensors co-located by the NSW Government (NSWG) with their reference PM_2.5_ monitors in Armidale, Orange, Gunnedah, and Muswellbrook (NSW) [23]. The spatial and temporal variation was assessed by installing some of the Armidale PA sensors at other locations when they were not co-located with the NSWG reference monitor (see Table 1), as well as from other publicly available PA data.

## 2. Materials and Methods

### 2.1. PA Data and Calibration Checks

**Figure A2:**
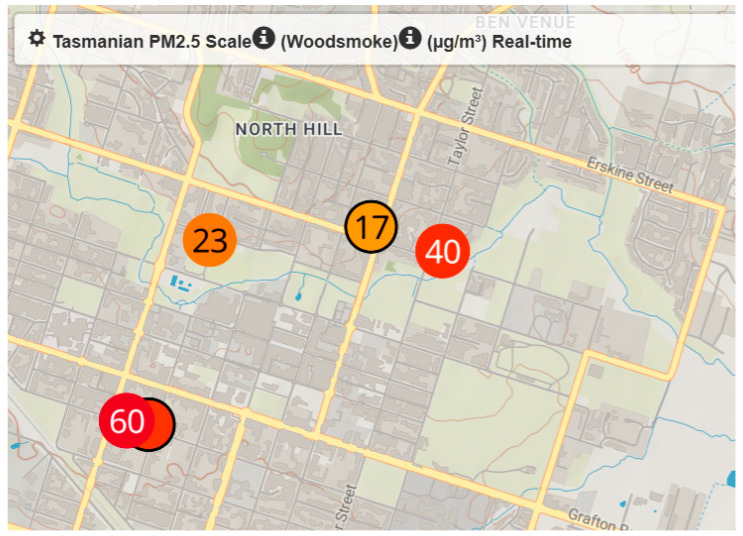
Screenshot of PA map showing PM_2.5_ pollution (woodsmoke conversion) at NSWG-A (PM_2.5_ = 23 μg/m^3^), outdoors at sites RS (60 μg/m^3^), RE (40 μg/m^3^), indoors at site RSi (red circle) and indoors at RCEi (17 μg/m^3^). Different colour scales are available to illustrate PM_2.5_ concentrations including the Tasmanian PM_2.5_ colour scale (shown here).

This project utilized PA sensors purchased by the Australian Air Quality Group and Sustainable Living Armidale in 2017 and the Armidale Regional Council (ARC) in 2018. After their formal calibration in 2018 [11], the three sensors installed in 2017 and four installed in 2018 were retained for continued use and calibration checks. The NSW Government Department for Planning and Environment (DPE) has also continuously co-located a PA sensor at their Armidale site (NSWG-A) since May 2019. Indoor and outdoor PM_2.5_ pollution has also been monitored continuously at a residential site in south Armidale (RS), as well as outdoor PM_2.5_ at a residential site in East Armidale (RE) from mid-2021, and inside a house in central/east Armidale (RCEi) from mid-2022. Table 1 shows dates and durations of sensor deployment, including co-location at NSWG-A. Figure A2 provides a map of the monitoring locations in Armidale.

Calibration drift was assessed by comparing daily average PA PM_2.5_ from 2019 to 2022 at the NSWG-A co-location site (based on calibrations using 2018 data, Table 1) with reference PM_2.5_ data from the NSWG Tapered Element Oscillating Microbalance (TEOM) [23], which uses the Filter Dynamics Measurement System (FDMS) to adjust for the loss of volatile compounds from the microbalance [11]. FDMS directs particle-laden air to the microbalance for 6 min (to weigh particles), then filtered air for the next 6 min (reference period, to measure the loss of volatiles)—a 12 min cycle repeated five times per hour. Because air pollution is measured for only half the time, any variation in pollution levels within each 12 min block increases the overall measurement error compared to the true pollution levels.

Data were downloaded as 2 min averages from the PA website and hourly averages calculated to match the TEOM PM_2.5_ data using the midpoint of the reported time interval (i.e., a record from 23:58:59 to 00:00:59 was counted in the hour from 23:00 to midnight). NSWG measurements ignore daylight saving time, so this analysis did likewise.

PA units contain two identical Plantower PMS 5003 laser sensors (‘A’ and ‘B’). Consequently, any problems are likely to show up as discordance between the ‘A’ and ‘B’ sensors. Minimal discordance was observed prior to 2019, but a series of spurious readings by one sensor in PA ARC1 from 12 noon to 19:00 (possibly caused by a speck of dirt that cleared itself) highlighted the need to check and adjust for discordance. Consequently, when the absolute difference (AD) between hourly averages for the ‘A’ and ‘B’ sensors from a PA unit exceeded 10 μg/m^3^ and also exceeded 50% of the mean (which happened for an average of 1.25 h per year per sensor), the hourly average was calculated as wt1 × L + wt2 × H, where L and H are the low and high PM_2.5_ measurements from the two sensors and wt1 = 0.5 × (1 + min(1, AD/mean(L,H))) and wt2 = (1 − wt1). Thus, H was not used if the difference between sensors exceeded their mean and also exceeded 10 μg/m^3^. Hourly measurements were then converted to daily averages, excluding any days with less than 23 hourly measurements. For PA sensors, two days (12 August 2019, 9 January 2020) were lost due to problems with the Wi-Fi system at NSWG-A. For TEOM data, a total of 91 days in 2019–2022 had less than 23 hourly measurements. However, 56 of the 91 had no ALT-34 data, or were in the Black Summer bushfire period [24], or the period from January to May 2022 (when there may have been problems with TEOM data—see Section 3.1), leaving an additional 35 days with less than 23 hourly TEOM measurements that were excluded from the final analysis.

As an additional check, readings were compared with those from the Ecotech (Aurora 1000G) integrating nephelometer (neph) at NSWG-A using a published relationship for woodsmoke [11]:

woodsmoke PM_2.5_ = 0.53 + 22.186 × neph (1)

Summary statistics comparing PA and TEOM PM_2.5_ measurements (for all days with at least 23 h of measurements) included correlations (r), root mean squared errors (RMSE), slopes of the relationships using reduced major axis (RMA) [25], and ordinary least squares (OLS) regression, as well as intercepts from the OLS regression.

### 2.2. Comparison with ALT-3.4

Plantower sensors report counts by particle size categories, including counts of particles with diameter >0.3, >0.5, >1, and >2.5 microns. Two proprietary inbuilt algorithms (cf1, based on the density of metal particles and cfa, based on the average density of atmospheric particles [26,27]) provide conversions of counts into PM concentrations (μg/m^3^). The research in Armidale to calibrate individual PA monitors (Table 1) and derive the PA woodsmoke conversion [11] also considered particle counts, but the high correlations between counts for the different particle size categories suggested that little would be gained for Armidale’s PM_2.5_ aerosol over an algorithm based on cf1. However, researchers in the USA [12] noted that the cf1 algorithm assigned concentrations below a threshold to zero (leading to potential underestimation at low PM_2.5_ levels). After comparing 5 years of daily PM_2.5_ data from 47 US Federal regulatory sites with 194 nearby PA monitors, an alternative algorithm to calculate PM_2.5_ directly from particle counts was proposed:

ALT-3.4 PM_2.5_ = W_0.5_ + W_1_ + W_2.5_ (2)

where W_0.5_, W_1_, and W_2.5_ represent the weights of particles in each size category, calculated as W_0.5_ = 3.4 × 0.00030418 × N1, W_1_ = 3.4 × 0.0018512 × N2, and W_2.5_ = 3.4 × 0.02069706 × N3, for N1 = the count of particles in the smallest size category (i.e., the count of particles of size > 0.3 microns minus the count for size > 0.5 microns) and similarly, N2 and N3 are counts of particles in the second smallest (0.5 to 1 micron) and third smallest (1 to 2.5 microns) size categories [12]. As explained in the published, peer-reviewed research [12], counts in each size category are multiplied by an empirically derived factor of 3.4 times 0.00030418, 0.0018512 and 0.02069706, respectively (representing the density of water particles in each size category) to convert numbers of particles to estimated weights.

The ALT-3.4 algorithm (requiring no prior calibration of individual PA units) was compared to TEOM PM_2.5_ in the same manner as the PA woodsmoke calibrations (Table 1).

### 2.3. NSW Government Monitoring Sites at Gunnedah, Orange, and Muswellbrook

The NSW Government also installed PA sensors at their sites in Gunnedah and Orange (where FDMS TEOMs measure PM_2.5_) and Muswellbrook (where PM_2.5_ is measured by a beta attenuation monitor, BAM). Muswellbrook’s PM_2.5_ originates from more diverse sources, including woodsmoke (for which the particle size distribution peaks at about 0.1–0.2 microns [28]) and mining dust (with many coarser particles between 2.5 and 10 microns). PM_10_ data were therefore downloaded for the NSWG reference stations to provide an indication of the amount and proportion of coarser particles in the aerosol. As well as comparing NSWG PM_2.5_ measurements with PA using the default woodsmoke and ALT-34 conversions (no calibration of individual PA units), the proportion (P1) of PM_2.5_ particles below 1 micron from the PA sensor was investigated as a potential indicator of particle sources and potential biases, i.e.,

P1 = (W_0.5_ + W_1_)/(W_0.5_ + W_1_ + W_2.5_) (3)

## 3. Results

### 3.1. Stability, Accuracy, and Spatial Variation in PurpleAir Measurements

**Figure 1:**
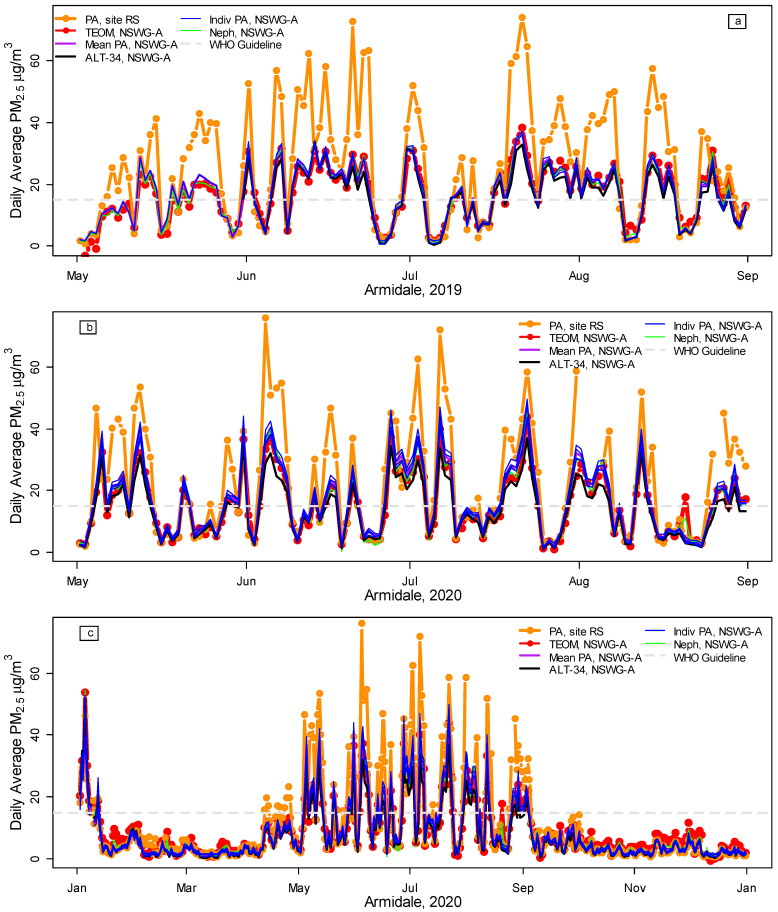
Illustration of the spatial variation in Armidale, 2019–2020, contrasting daily average PurpleAir (PA) PM_2.5_ at site RS with PM_2.5_ measurements at the NSW Government site (NSWG-A) in winter 2019 (**a**), winter 2020 (**b**), and all year in 2020 (**c**). The graphs show the PA woodsmoke calibration (Table 1) at site RS (orange lines) vs. FDMS TEOM PM_2.5_ (red), the average of PA woodsmoke PM_2.5_ for all co-located sensors at NSWG-A (purple, details in Table 1), the average ALT-34 PM_2.5_ (black, all available data for co-located monitors), individual PA woodsmoke PM_2.5_ measurements (blue), nephelometer data converted to PM_2.5_ using Equation (1) (green), and the WHO daily air quality PM_2.5_ guideline (maximum daily average of 15 μg/m^3^). The high PM_2.5_ in January 2020 was due to the Black Summer bushfires [24].

**Figure 2:**
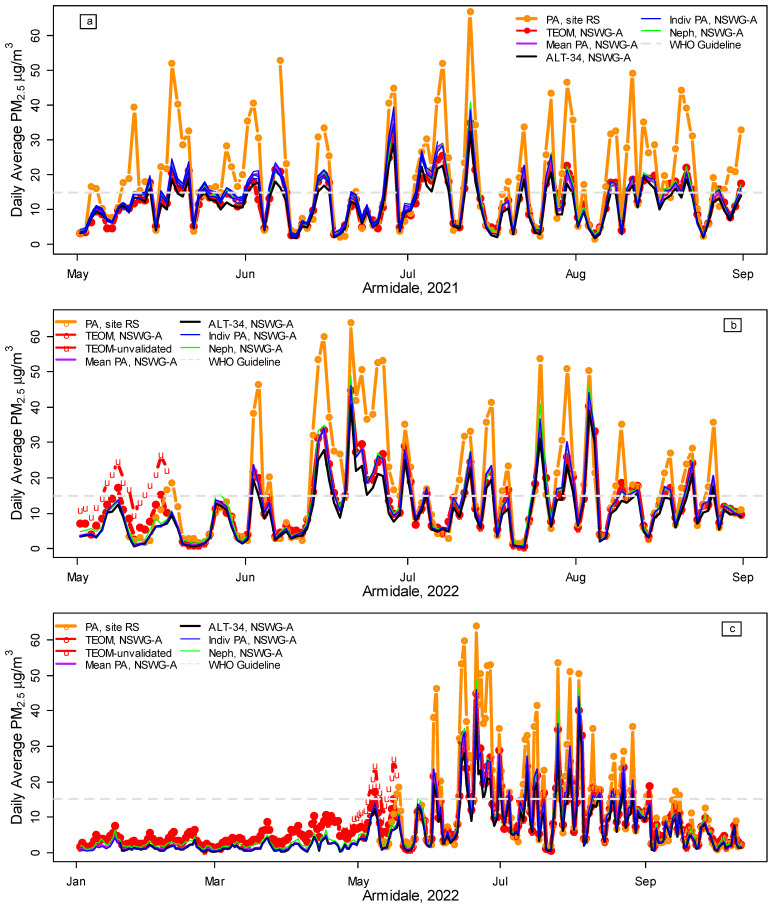
Illustration of the spatial variation in Armidale, 2021–2022, contrasting daily average PurpleAir (PA) PM_2.5_ at site RS with PM_2.5_ measurements at the NSW Government site (NSWG-A) in winter 2021 (**a**), winter 2022 (**b**), and all year in 2022 (**c**). The graphs show PA woodsmoke (Table 1) at site RS (orange lines) vs. PM_2.5_ measurements at NSWG-A comprising FDMS TEOM (red lines), the average of all PA woodsmoke PM_2.5_ for co-located sensors at NSWG-A (purple, details in Table 1), the average ALT-34 PM_2.5_ (black, all available data for co-located monitors), individual PA woodsmoke PM_2.5_ measurements (blue), nephelometer data converted to PM_2.5_ using Equation (1) (green) and the WHO daily air quality PM_2.5_ guideline (maximum daily average of 15 μg/m^3^). TEOM measurements prior to validation (24 April to 17 May 2022) are denoted by ‘u’ in Figure 2b,c.

Figure 1 and Figure 2 illustrate the spatial variation in PM_2.5_ in Armidale, comparing the daily average PM_2.5_ at site RS (orange lines) with site NSWG-A in 2019, 2020, 2021, and 2022 (to 12 October). Figure A2 provides a map of the locations. All PM_2.5_ measurements at the NSWG-A co-location site show good agreement (Figure 1 and Figure 2, Table 2), with almost identical lines for TEOM (red), the average of all the PA woodsmoke PM_2.5_ (purple, dates, and calibration/conversion details in Table 1) and nephelometer data converted to PM_2.5_ using Equation (1) (green). The blue lines show PM_2.5_ from individual PA units when co-located at NSWG-A. The discrepancies between monitoring equipment are relatively small compared to the large spatial variation, exemplified by the difference between site RS (orange lines) and NSWG-A (other colors). Despite requiring no prior calibration of the PA units, the ALT-34 conversion (black lines) also performs well.

However, when the preliminary data for the TEOM were downloaded on 13 October 2022, a substantial discrepancy was noted between the preliminary unvalidated TEOM data and the PA PM_2.5_ for late April to mid-May 2022 (unvalidated data are labeled ‘u’ in Figure 2b,c). The NSW Government provides preliminary data online after an initial automated validation procedure. Full validation is carried out over the following months [29]. When the fully validated data became available, the values for 24 April to 17 May were much closer to the PA PM_2.5_. This suggests that PA sensors may have a useful role (perhaps as part of a more sophisticated preliminary validation procedure) in quickly identifying potential problems, such as the discrepancies between the initial and validated TEOM data for 24 April to 17 May 2022.

Figure 2, in fact, suggests the problem might have extended back to January 2022, so the period from January to May 2022 was not included in the final analysis. However, the correlation, r = 0.97, between all PA woodsmoke and TEOM PM_2.5_ data (1 January 2019 to 12 October 2022) was almost identical to the r = 0.98 shown in Table 2. Omitting the period of the problematic TEOM data (January–May 2022) and the Black Summer bushfires (November 2019 to February 2020 [24]) therefore made little difference to the overall conclusions.

Summary statistics on the relationship between the TEOM and PA (woodsmoke and ALT-34) PM_2.5_ are shown in Table 2. To ensure the comparability of results for woodsmoke and ALT-34, the main analysis for Armidale was restricted to periods with both ALT-34 and woodsmoke PM_2.5_ data (31 May 2019–12 October 2022), excluding the Black Summer bushfires [24] and January to May 2022, when there may have been problems with the accuracy of the TEOM PM_2.5_. The correlations (r) between the PA and TEOM PM_2.5_ were 0.98 for both PA woodsmoke and ALT-34. The RMA regression slope of 0.91 suggests that the PA woodsmoke measurements slightly over-estimated the TEOM PM_2.5_ at high pollution levels, whereas the ALT-34 slightly underestimated them (RMA slope 1.03). The low RMSE for the PA woodsmoke for winter (June–August) 2022 of 1.41 μg/m^3^ demonstrates that, even during the fourth year of operation, the PA measurements were still highly accurate, as does the RMSE of 2.03 μg/m^3^ for all 980 days (31 May 2019–12 October 2022) with valid TEOM and woodsmoke PM_2.5_ data from all PA units at NSWG-A.

### 3.2. PA vs. TEOM Measurements at Orange and Gunnedah

**Figure 3:**
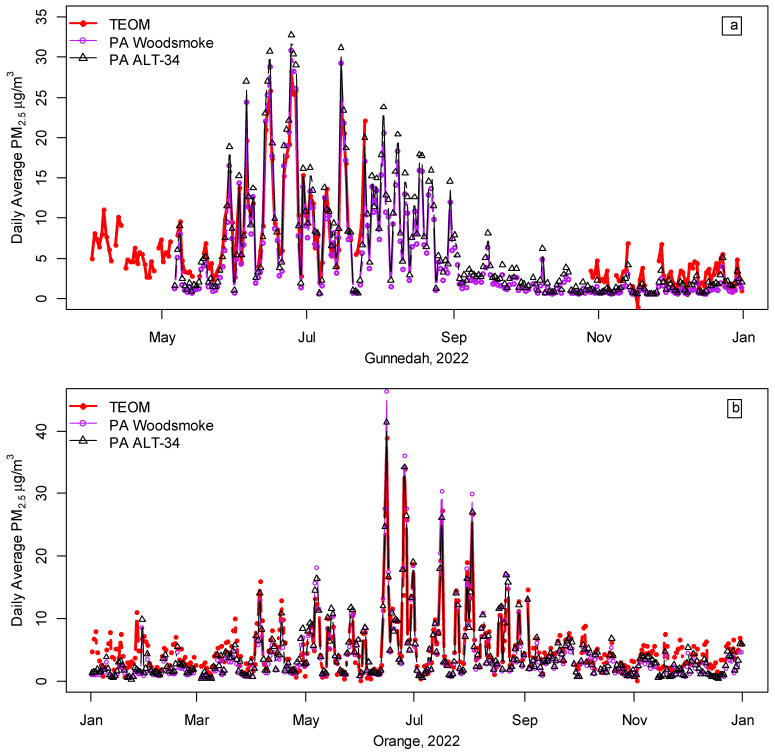
Comparison of publicly available NSWG data for PM_2.5_ (FDMS TEOM) and PurpleAir (PA) PM_2.5_, woodsmoke, and ALT-34 calibrations, from co-located sensors in winter 2022 at Gunnedah (**a**) and Orange (**b**).

Figure 3 and Table 2 compare all publicly available PA data in 2022 for the co-located monitors at Orange and Gunnedah with daily average TEOM PM_2.5_ measurements (all available hourly averages in 2022 were downloaded from the NSWG website [23] and converted to daily averages for all days with at least 23 h of measurement). The average TEOM PM_2.5_ from April to September in Gunnedah (10.1 μg/m^3^) and Orange (6.6 μg/m^3^) were lower than in Armidale (13.2 μg/m^3^), but the correlations of r = 0.97 to 0.98 between the TEOM PM_2.5_ and both the woodsmoke and ALT-34 calibrations were similar to those observed in Armidale, as were the RMSE of 1.4 to 2.6 μg/m^3^ (Table 2). For Gunnedah, the high correlation (r = 0.98) for May–July 2023 suggests that PM_2.5_ from the co-located PA sensor could (after further checking and analysis) serve as a useful approximation for Gunnedah’s missing TEOM data (26 July onwards).

In contrast to the wood heating season, the PA sensors underestimated PM_2.5_ in the warmer months. Inspection of Gunnedah’s PM_10_ and PM_2.5_ data from the NSWG reference monitor showed that, except for a monthly average of 3.0 μg/m^3^ in July 2022, monthly averages for coarser particles (2.5 to 10 microns) ranged from 4.3 to 6.0 μg/m^3^ for all days with at least 23 hourly measurements, while PM_2.5_ monthly averages ranged from 2.8 μg/m^3^ (November and December) to 13.6 μg/m^3^ (June). Particle mass is proportional to the cube of the diameter, i.e., particles 2.5 microns in diameter weigh about 16 times more than those with a diameter of 1 micron, so when the aerosol mass is skewed towards the larger end of the size distribution, algorithms based on a typical size distribution for combustion particles (including those used by PA sensors) are likely to underestimate particle mass. Although both the woodsmoke and ALT-34 calibrations underestimated PM_2.5_ during the summer months, the year-round correlation of r = 0.94 indicates that the underestimation in warmer months is relatively unimportant.

The results for Orange were similar to those for Gunnedah; both the woodsmoke and ALT-34 calibrations were highly correlated with the TEOM PM_2.5_ (r = 0.97 to 0.98) from April to September with a low RMSE of 1.4 to 1.7 μg/m^3^ (Table 2). For the year as a whole, the correlations (r = 0.94) were lower and the RMSE (1.91 to 2.22 μg/m^3^) were slightly higher than for April to September. Thus, for both Orange and Gunnedah, PA measurements provided a good approximation of the TEOM PM_2.5_ in winter with some underestimation from October to March.

### 3.3. PA vs. BAM PM_2.5_ Measurements in Muswellbrook

**Figure 4:**
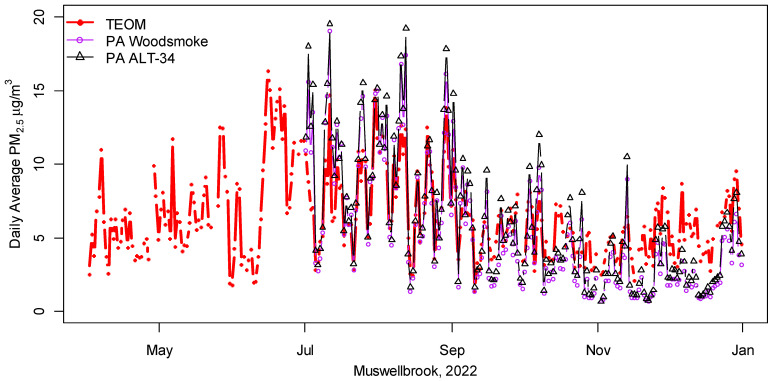
Comparison of publicly available TEOM and PurpleAir (PA) measurements at Muswellbrook.

A study of the PM_2.5_ aerosol at Muswellbrook from January to December 2012 [30] used chemical analyses to show that it comprises wood heater smoke (30%), secondary sulphate and nitrate (23%), sea salt including industry-aged sea salt (16%), biomass smoke (e.g., from wildfires and hazard-reduction burns) 12%, soil dust (11%), and vehicle/industry emissions (8%) [30]. Despite this diversity, Figure 4 and the correlations from April to September of r = 0.89 between the PA woodsmoke and the co-located BAM PM_2.5_ (RMSE: 2.27) and r = 0.86 year-round (RMSE: 2.48) demonstrate the accuracy of the PA measurements.

As noted for Orange and Gunnedah, PA measurements at Muswellbrook tended to underestimate the BAM PM_2.5_ during the warmer months, when a higher proportion of particles originate from windblown dust. The size distributions of both woodsmoke particles and motor vehicle traffic peak at about 0.1–0.2 μm [28,31], suggesting that the proportion of particles in the three PA size categories (<0.5, 0.5–1, and 1–2.5 μm) might provide additional useful information to discriminate between smaller particles from vehicle and smoke emissions and larger dust particles. P1, the estimated proportion of the PA ALT-34 particles up to 1 μm (Equation (3)), was positively correlated with the residuals (BAM PM_2.5_ − PA, r = 0.64 for ALT-34 and r = 0.62 for woodsmoke), implying that a higher proportion of particles below 1 μm increased the likelihood that PA sensors would underestimate Muswellbrook’s PM_2.5_ measurements. In contrast, the ratio PM_2.5_/PM_10_ was negatively correlated with the residuals, implying that low PM_2.5_/PM_10_ ratios (which generally indicates that coarser dust particles predominate, i.e., outside the wood heating season) also increase the likelihood that PA measurements will underestimate PM_2.5_. A US study [32] has now also evaluated the performance of PA sensors in different types of aerosols and proposed a dust correction factor when the ratio of 0.3 μm to 5 μm particle counts from the PA sensor is below 190 [32]. Another possibility is to integrate data from low-cost sensors and satellite remote sensing [33,34] to help identify and discriminate between emissions from local urban aerosols and smoke and dust plumes.

Overall, the relatively high year-round correlations for the woodsmoke and ALT-34 conversions of, respectively, r = 0.86 and 0.85, and RMSE of 2.49 and 2.58 (for brevity, Muswellbrook’s statistics were not included in Table 2) suggest that appropriately converted PA data provide highly useful PM_2.5_ measurements. The ability to measure PM_2.5_ in many different locations for a very modest cost, including inside our homes, is an attractive feature of PA.

### 3.4. Indoors vs. Outdoors

**Figure 5:**
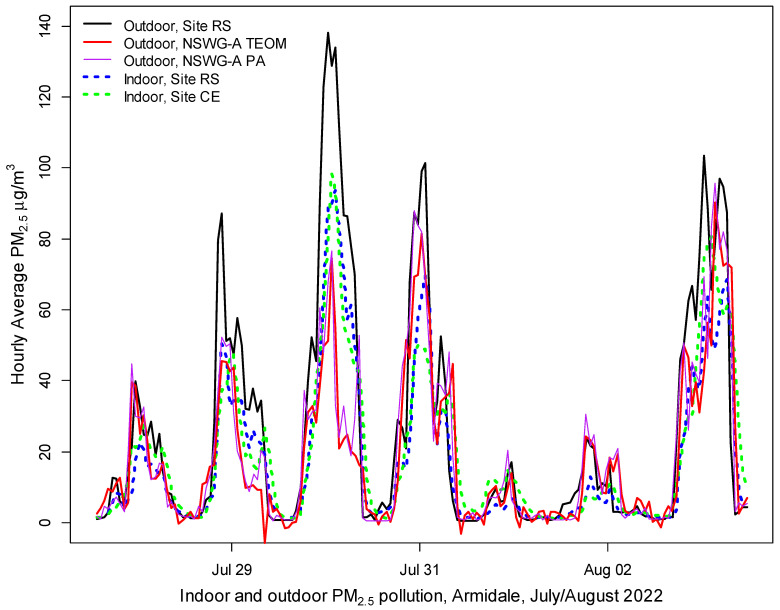
Comparison of indoor measurements inside two houses in south and central Armidale (sites RS and CE, dotted lines) compared with outdoors (site RS and NSWG-A), Armidale 2022.

Figure 5 shows the indoor and outdoor measurements at the Armidale RS site, indoor measurements in a house in central Armidale, and the PA and TEOM measurements at NSWG-A, which was less polluted than inside either house. However, the outdoor PM_2.5_ at site RS was even higher than the PM_2.5_ inside either house. The graph confirms other research that, depending on the ‘leakiness’ of the home and the frequency of opening doors and using open flued heaters (that draw air into the house to replace air leaving the flue), outdoor smoke levels can penetrate indoors within 1 to 2 h. PA measurements can therefore provide a useful indicator of the woodsmoke infiltration rates without formal blower tests. These measurements support the results of other research showing that staying indoors during smoke events provides limited protection without HEPA filtration [35].

### 3.5. Comparison of 1-Day Averages at Selected Sites in Armidale, NSW, Canberra (ACT), and Christchurch (NZ)

**Figure 6:**
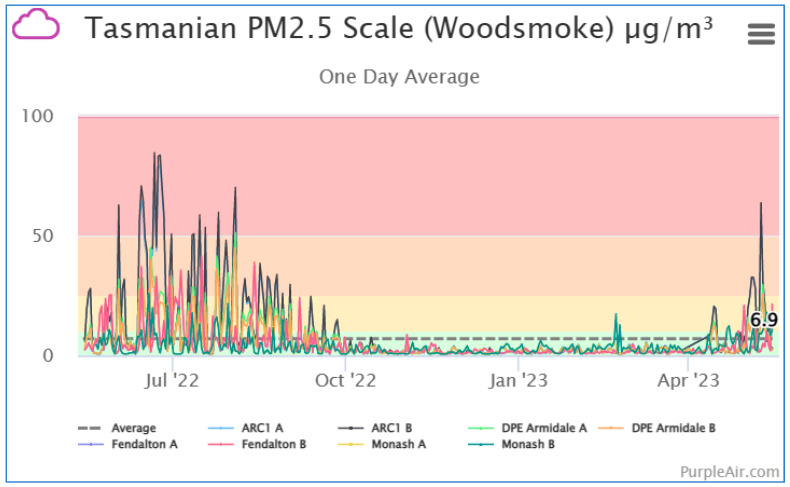
Example of one-day average measurements from the PurpleAir website, showing sites RE (ARC1 in the legend) and NSWG-A (DPE Armidale), outdoor measurements at house in Monash, ACT, and Christchurch (Fendalton, NZ). The graph shows separate PM_2.5_ measurements from the ‘A’ and ‘B’ laser sensors in each PA unit using the woodsmoke conversion.

Data from all the public PA monitors on the PA website (map.purpleair.com, accessed on 1 July 2023) are available as graphs of selected sites either in real time, 10 min, 30 min, 1 h, 6 h, 1-day, 1-week, or 1-month averages. Figure 6 shows PM_2.5_ (woodsmoke calibration) for the NSWG-A and RE sites in Armidale, together with a PA monitor at a residence in Monash, ACT, and the only PA monitor with publicly available data in Christchurch (Fendalton, about 4 km from English Park, St Albans, where PM_2.5_ is measured by Environment Canterbury and displayed on the Land Air Water Aotearoa website [36]). All show high PM_2.5_ levels during the wood heating season and low PM_2.5_ at other times of the year.

## 4. Discussion

### 4.1. Accurate, Comprehensive Measurements with Minimal Calibration Drift

The results from co-located PA monitors with the NSWG-A TEOM confirm that the woodsmoke calibration/conversion provided accurate measurements of the PM_2.5_ pollution in Armidale with minimal calibration drift over 4 years. These results add to the growing body of evidence of the utility and accuracy of calibrated PA sensors, and the lack of evidence of calibration drift [13,37]. This study also confirmed the similarity of the woodsmoke and ALT-34 conversions. The year-round correlations of r = 0.94 (Gunnedah and Orange) and 0.98 (Armidale) are comparable to the correlations between PM_2.5_ measurements of co-located US Federal Reference (FRM) and Federal Equivalence (FEM) PM_2.5_ methods: r = 0.98 (FRM-FRM), 0.96 (FRM-FEM), and 0.91 (FEM-FEM) [13].

In this study, comparisons were based on daily averages for the PA and reference data with valid measurements for at least 23 h per day—a stricter requirement than the standard practice of reporting averages for days when at least 18 h of data are available. The correlations between two sets of measurements depend on the accuracy of both. It is therefore important to note that, because of the substantial temporal variation in woodsmoke-affected areas, an 18 h average excluding 6 pm to midnight could be very different from an 18 h average for the same day excluding 10 am to 4 pm. Even the fact that FDMS TEOMs measure PM_2.5_ for only 30 min in each hour can lead to inaccuracies if there is substantial variation over short periods of time. For example, on 21 June 2023, the average TEOM PM_2.5_ at NSWG-A for 7–8, 8–9, 9–10, 10–11 am was 20.9, 65.0, 53.0, 20.7 μg/m^3^, respectively. The co-located PA sensor showed substantial variability in PM_2.5_ levels within each hour, suggesting that some of the variation in the TEOM PM_2.5_ over this 4 h period might have been influenced by whether the TEOM measurement phases coincided with periods of above or below average PM_2.5_ pollution.

Negative pollution measurements indicate inaccuracy, e.g., the TEOM reading of −5.7 μg/m^3^ from 8 to 9 am on July 29 (Figure 5) was at variance with the small increases in all the PA sensors and the nephelometer. Although errors are reduced by averaging, even daily average TEOM PM_2.5_ can be negative; there were 42 days between October 2018 and March 2021 when Armidale’s validated 24 h average TEOM PM_2.5_ pollution was below zero. Only four occurred after 30 May 2019 (the period used for the analysis shown in Table 2); they were retained in the analysis.

Some interesting observations about fine particle measurements were noted for the equipment used in Tasmania including optical particle counters [38] and the TEOMs used in NSW prior to 2012 (see Appendix B). Use of optical particle counters to measure PM_2.5_ pollution from woodsmoke gained widespread acceptance in Tasmania following studies confirming the accuracy of the BLANkET network of medium-cost DustTrak monitors in measuring woodsmoke PM_2.5_ [39]. These results formed the basis of the study of the health effects and costs of landscape fires and wood heater pollution in Tasmania [21]. In Armidale, until the end of winter 2017, woodsmoke PM_2.5_ pollution was also measured by a TSI DustTrak 8520 using the Tasmanian BLANkET calibration. Co-locating a PA monitor with Armidale’s DustTrak in 2017 showed that DustTrak and PA measurements (using their respective woodsmoke calibrations) were almost identical [11].

A common criticism of optical PM_2.5_ sensors is that water droplets in mist and fog could produce spuriously high readings, although this can be avoided by warming the airstream to vaporize any water droplets before entry to the particle counters [9,16]. A small amount of heat is generated by the PA Wi-Fi unit, so temperatures inside PA sensors are already above ambient; this probably explains why there was no need to correct for relative humidity (RH) measurements in Armidale [11], although a study by the US EPA [15] recommended a small adjustment:

USEPA PM_2.5_ = 0.524 × cf1 − 0.0862 × RH + 5.75

The latter two terms equate to an intercept of −2.87 at 100% RH, an intercept of zero at 66.7% RH, and an intercept of 2.91 at 33% RH [15]. For large amounts of bushfire smoke, an additional quadratic term in the conversion equation was recommended for cf1 > 343 μg/m^3^ [14]. In recognition of the utility of the PA measurements in the USA, converted PA data are now published on the government website: fire.airnow.gov (accessed on 13 November 2023).

The paucity of government monitoring sites in regional Australia compared to PA sensors indicates that, in many cases (especially in winter and during bushfires), readings from nearby PA sensors will provide useful additional measurements of the PM_2.5_ pollution. Australian health protection and air pollution websites should therefore consider displaying appropriately converted PA measurements to supplement the data obtained from reference instruments. As long as community monitors are labeled appropriately, and the variation between nearby monitors is readily apparent, this information could be of considerable additional value, particularly in identifying pollution hotspots and characterizing population exposure.

An important advantage of PA sensors is that, as well as averages over periods of time from 10 min to 1 year, the PA website provides maps with real-time data and graphs of the previous 48 h, allowing an assessment of the current PM_2.5_ pollution and potential future PM_2.5_ pollution to guide personal choices. On 24 July 2022, for example, Armidale’s TEOM measurement was 2.5 μg/m^3^ PM_2.5_ from 5–6 pm, 32.4 μg/m^3^ from 6–7 pm, and 49.1 μg/m^3^ from 7–8 pm. Without the PA data, only the 2.5 μg/m^3^ would have been reported until about 7:20 pm, by which time, 49.1 μg/m^3^ (the TEOM PM_2.5_ from 7–8 pm) would be much closer to what people were breathing.

Indeed, because of the high spatial variation in woodsmoke PM_2.5_ and the accuracy of PA sensors demonstrated by this research, PA PM_2.5_ should be closer to what people are breathing in any particular woodsmoke-affected area of a town or city than measurements at a reference monitor some distance away. PA units can be installed inside homes to measure indoor pollution, or under a veranda or porch to measure the corresponding outdoor levels. If there is no Wi-Fi or mains power, solar power and the internal SD card, or a mobile phone hotspot, can be used to log the data. The importance of small scale variation was demonstrated by the consistent variation recorded by EPA Tasmania between five inter-calibrated sensors located on different sides of a single house [40]. The small size and low cost of PA units (AUD 275 each, including postage, for those installed in Armidale in 2017) contrasts with the NSWG-A’s air-conditioned monitoring site in Armidale that was set up in 2018 on several square metres of land at a cost of almost AUD 200,000 with annual operating costs exceeding AUD 30,000 [41]. Given estimated pollution costs exceeding AUD 10,000 per wood heater per year in Armidale [19] and Greater Sydney [20], indoor and outdoor measurements from PA (or other accurate low-cost sensors) can have a valuable role monitoring PM_2.5_ and assisting regulatory authorities.

### 4.2. Potential to Consider Other Particle Sizes

Optical particle counters that satisfy the US PM_2.5_ Federal Equivalence Method (FEM), such as the GRIMM EDM 180, use many size channels to convert particle counts to particle mass. The PA sensors were originally chosen for Armidale because of their extremely high correlations with the GRIMM and BAM measurements in the field evaluation tests of the co-located monitors conducted by AQ-Spec in 2016 and 2017 [42] (r = 0.99 for 24 h GRIMM PM_2.5_ and PM_1_; r = 0.98 for 24 h BAM PM_2.5_, 1 h GRIMM PM_2.5_ and PM_1_), these being the highest field correlations of all the low-cost monitors tested [42].

Studies comparing PM_1_ and PM_2.5_ suggest that PM_1_ might be even more closely related to adverse health impacts than PM_2.5_ [43,44,45,46,47,48]. The strong correlations between PA PM_1_ and GRIM PM_1_ in the AQ-Spec tests of r = 0.98 and 0.99, respectively for 1 h and 24 h PM_1_ with average regression slopes of 0.75 (1 h) and 0.76 (24 h) suggest that PA measurements could be used to supplement the limited PM_1_ measurements available in Australia to help evaluate the relationship between PM_1_ and adverse health impacts.

### 4.3. Enhancing Estimates of Population Exposure, Costs, and Health Impacts

Accurate air quality measurements are important, because of the large spatial variation in PM_2.5_ pollution from wood heaters observed in many studies [6,7,8,49] and the need to “fully account for this for valid population exposure estimates” [50]. Additional information on spatial variation can help improve the estimates of population exposure and reduce the potential downward bias in estimates of exposure–response functions derived from ecological epidemiology studies [25]. Thus, when wood heaters or landscape fires are the dominant source of PM_2.5_ pollution, information on spatial and temporal variation from low-cost sensors can enhance the overall accuracy of the estimates of population exposure, consequent health impacts and costs of the pollution.

Two major studies of the health costs of PM_2.5_ pollution in Greater Sydney estimated the population-weighted exposure to human-made PM_2.5_ pollution (PWE-PM_2.5_) and premature deaths by modeling pollution concentrations from emissions inventory data combined with PM_2.5_ measurements. The first, published in 2020, studied 1 July 2010–30 June 2011 [51]. The second, published in 2023, studied the calendar year 2013 [20]. The large discrepancy between the estimates for the wood heater pollution from the two studies (2013: 269 premature deaths, PWE-PM_2.5_ of 1.261 μg/m^3^, 42% of all PWE-PM_2.5_ vs. 2010–11: 100 premature deaths, PWE-PM_2.5_ of 0.49 μg/m^3^, 24% of all PWE-PM_2.5_) illustrates the potential for PM_2.5_ measured by calibrated PA and other community sensors to improve our understanding of spatial variation and the accuracy of different monitoring and modeling techniques, and so enhance overall accuracy.

As an indication of the need for multiple measurement techniques in multiple locations, and the potential role of low-cost sensors to ensure the most accurate estimates of population exposure, health impacts, and the costs of air pollution, Appendix B summarizes the procedures used in Tasmania and examines the accuracy of the PM_2.5_ measurements that led to the initial estimate of 100 of premature deaths from wood heater pollution in Greater Sydney.

### 4.4. Application to Other Situations and Other Types of Low-Cost Sensors

Wood heater pollution can vary substantially over periods of less than an hour and distances of a few tens of metres, whereas smoke pollution from bushfires can travel long distances. Even a widely spaced network of PA sensors in local towns could therefore provide accurate measurements (‘ground truth’) to complement satellite data on bushfire smoke, noting that PM_2.5_ estimated from satellite measurements of aerosol optical depth (AOD) has a spatial resolution of 500 m to 1 km [52] and temporal resolution determined by the frequency of satellite passes over the location.

Other researchers have integrated low-cost sensor and satellite data to provide NO_2_ measurements [53]. A NZ study [22] reported substantial annual costs of PM_2.5_ emissions from wood heaters (NZD 4.06 billion, equivalent to NZD 7550 per wood heater per year) and even higher costs of NO_2_ emissions (NZD 9.5 billion). The estimated health impacts of NO_2_ were noted to be higher than those reported by many previous studies. However, because of limited NO_2_ data, NO_2_ concentrations were estimated by modeling vehicle emissions [54]. Consequently, the authors suggested that the estimated effects of NO_2_ from their models could be a proxy for other traffic-related pollutants [54]. In Auckland, diesels (which emit PM_2.5_ and PM_1_ and NO_2_) accounted for 64% of emissions, with heavy diesels (representing only 4% of the fleet) contributing 61% of emissions [22]. The concentrations of black smoke, PM_2.5_, NO_2_, and benzene were reported to decrease to background concentrations within 100–150 m of a roadway [55]. A network of PA and NO_2_ sensors, perhaps supplemented by satellite data, could therefore assist in reconciling the results of the NZ study with other published studies.

## 5. Conclusions

The woodsmoke and ALT-34 calibrations of the PA sensors at the NSW Government monitoring site in Armidale were almost identical to the reference TEOM PM_2.5_ data. The minimal calibration drift over the 4 years of this study implies that even older PA sensors can provide useful population exposure information, provided appropriate checks are used, e.g., the consistency of the ‘A’ and ‘B’ sensors in each PA unit. The high spatial variation in ambient PM_2.5_ concentrations in areas affected by woodsmoke implies that an appropriately calibrated PA sensor located close to an area of interest may provide more representative PM_2.5_ measurements than a NATA-accredited instrument located even 100 metres away from that area. Similarly, at times when PM_2.5_ increases rapidly, residents may benefit from real-time air pollution data for the current hour [56], which can be provided by a low-cost sensor network.

In Tasmania, the study of the health effects and costs of landscape fires and wood heater pollution was facilitated by widespread acceptance that the BLANkET network of DustTraks provides accurate measurements of woodsmoke PM_2.5_. Australian health and air pollution sites should therefore consider displaying appropriately calibrated PA measurements to inform the public and supplement government air pollution data. Especially when several sensors are deployed in a particular area, they can provide useful information on spatial and temporal variation and population exposure to air pollution. For example, population-weighted PM_2.5_ exposure data and the associated financial costs of wood heater pollution in Armidale in 2018 were derived using autovariogram kriging to interpolate the PA and TEOM PM_2.5_ measurements to the Australian Bureau of Statistics population meshblocks [19].

In summary, the real-time PM_2.5_ measurements from PA sensors can help increase public awareness of PM_2.5_ pollution, provide evidence of unhealthy pollution levels in specific locations, and assist with the calculation of the population-weighted estimates of exposure to improve estimates of exposure–response functions and calculate health costs. When combined with cost–benefit analyses, such information will help inform public policy for managing local air quality and controlling emission sources.

## Figures and Tables

**Table 1:** Details of PurpleAir units located at the NSW Government site (NSWG-A) and residential sites in Armidale.

PurpleAir Unit ^1^	Dates at NSWG-A	Dates at RS, RSi, RE and RCEi ^2^	Calibration, 2018 ^3^
DPE, 29949	31 May 2019 onwards		0.53, 0.55 (D)
ARC2, 10476	1 January 2019–7 July 2021	RSi: from 7 July 2021	1.41, 0.524
ARC1, 10452	24 August 2019–7 July 2021	RE: from 20 July 2021	1.15, 0.516
ARC02, 10472	7 July 2021–27 July 2021	RSi: until 7 July 2021	0.53, 0.55 (D)
ARC17, 10140	27 July 2021–27 May 2022	RCEi: from 17 June 2022	1.66, 0.523
Arm1, 1732	1 January 2019–7 July 2021	RS: from 7 July 2021	0.53, 0.580
Arm2, 1738	7 August 2021–22 May 2022		0.53, 0.510
Arm3, 1720	7 July 2021–7 August 2021	RS: 1 January 2019–7 July 2021	0.53, 0.596

^1^ PurpleAir (PA) units are identified by their name and unique sensor number. ^2^ RS, RSi = outdoors and indoors at site RS; RE = outdoors, site RE; RCEi = indoors, site RCE. See Figure A2 for a map of locations. ^3^ Calibrations (intercept, slope in the equation: TEOM = intercept + slope × cf1, where cf1 is the PA cf1 value) were based on 2018 data [11]. No calibrations were performed for ARC02 and DPE, so the default woodsmoke conversion (D) = 0.53, 0.55 on the PA website (map.purpleair.com, accessed on 21 November 2023) was used.

**Table 2:** Summary statistics for the relationships between daily average TEOM FDMS PM_2.5_ measurements and averages of all co-located PA sensors (woodsmoke and ALT-34 calibrations/conversions, as described in Table 1 and Equation (2)) for Armidale (NSWG-A), Orange, and Gunnedah, for April to September (months when wood heating is used), as well as all available valid data, and winter (June to August) 2022 in Armidale.

Comparison Period	PA Calibration/Conversion	Correlation (r ± SE), PA vs. TEOM	RMSE ^1^	Days Compared	RMA Slope	OLS Regression ^2^		Mean TEOM	Mean PA
						Slope	Intercept		
***Armidale, 31 May 2019 to 12 Oct 2022 *** **^3^**									
April–September	woodsmoke	0.98 ± 0.01	2.09	608	0.92	0.90	1.29	13.16	13.24
	PA-ALT-34	0.98 ± 0.01	2.47	608	1.06	1.04	1.18	13.16	11.56
All valid data in all years	woodsmoke	0.98 ± 0.01	2.03	980	0.91	0.90	1.41	9.90	9.48
	PA-ALT-34	0.98 ± 0.01	2.31	980	1.03	1.01	1.44	9.90	8.35
June–August 2022	woodsmoke	0.99 ± 0.02	1.41	92	0.95	0.94	0.85	14.45	14.47
	PA-ALT-34	0.99 ± 0.02	2.69	92	1.12	1.11	0.75	14.45	12.35
***Orange, 2022***									
April–September	woodsmoke	0.97 ± 0.02	1.74	176	0.88	0.86	1.27	6.57	6.17
	PA-ALT-34	0.98 ± 0.02	1.40	176	0.96	0.94	0.66	6.57	6.30
All year	woodsmoke	0.94 ± 0.02	2.22	352	0.87	0.82	1.98	5.28	4.02
	PA-ALT-34	0.94 ± 0.02	1.91	352	0.93	0.88	1.49	5.28	4.29
***Gunnedah, 2022***									
April–September	woodsmoke	0.98 ± 0.03	2.41	75	0.82	0.80	2.85	10.14	9.12
	PA-ALT-34	0.98 ± 0.03	2.58	75	0.77	0.75	2.38	10.14	10.32
All year	woodsmoke	0.94 ± 0.03	3.33	189	0.83	0.78	3.31	6.74	4.42
	PA-ALT-34	0.94 ± 0.03	2.97	189	0.78	0.73	2.86	6.74	5.32

^1^ RMSE = root mean square error, i.e., sqrt(mean(PA-TEOM)^2^); ^2^ fitting TEOM = PA × slope + intercept; ^3^ excludes the Black Summer bushfires (November 2019–January 2020 [24]) and January–May 2022 (see Section 3.1).

**Figure A3:**
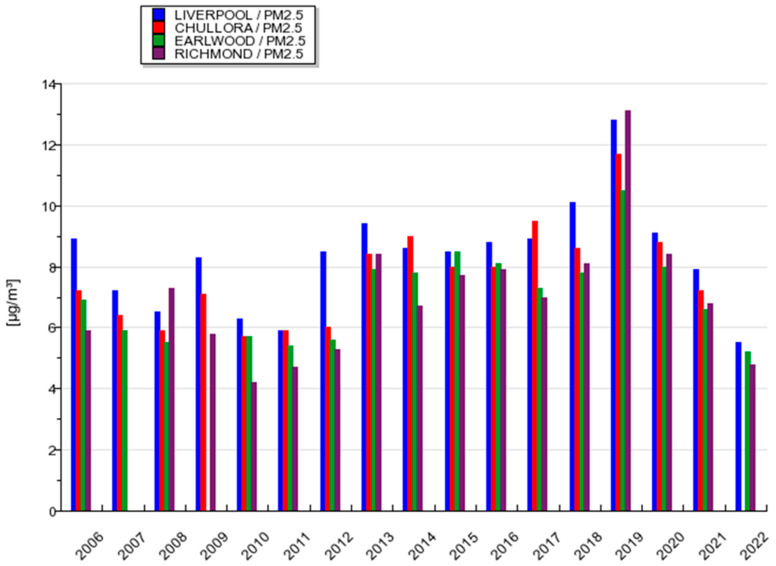
Bar chart of PM_2.5_ measurements at Liverpool, Chullora, Earlwood, and Richmond, Sydney (NSW government data [23]).

**Figure A4:**
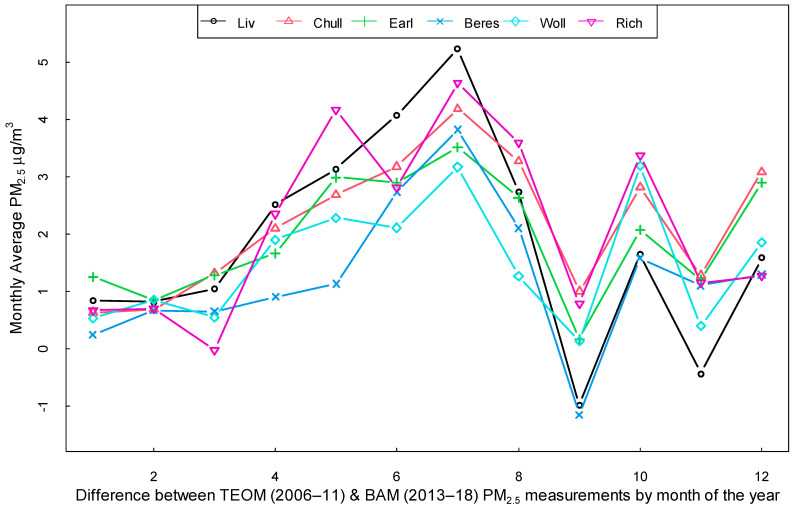
Difference between TEOM (2006–2011) and BAM (2013–2018) measurements by month of the year at Liverpool, Chullora, Earlwood, Beresford, Wollongong, and Richmond (source: NSW Government data download facility [23]).

## Data Availability

The daily averages for Armidale, Gunnedah, Orange and Muswellbrook are available at https://www.dropbox.com/sh/7rxb1d6xhecdo1d/AABTDiYRn5svUNptP1EXEz4Za (accessed on 13 November 2023). The raw data are available via download facilities on the PA and NSWG websites.

## Appendix B. Benefits of Multiple Measurement Techniques and Locations to Assess Population Exposure

EPA Tasmania’s several years of experience with a small optical particle counter (TSI 8520 DustTrak) allowed their researchers to conclude that relatively low-cost, low-power instruments could give a very good indicative measurement of Tasmania’s PM_2.5_. This led to the development of the BLANkET system [58], initially with 15 PM_2.5_ monitoring stations, increasing to 29 by 2015 [39]. Further developments included car-based PM_2.5_ measurements (Travel BLANkET [8]), an even lower cost portable system based on the Dylos DC-1700 laser particle counter (Baby BLANkET [16]), then, a system using Plantower sensors [40].

Research by the Department of Primary Industries Water and Environment, Tasmania (DPIWE, a predecessor of EPA Tasmania), also highlighted the problems experienced with woodsmoke particle mass measurements using TEOMs prior to the FDMS system. DPIWE researchers derived a PM_10_ correction factor of (2.2-0.8T) for daily average temperatures, T, between 0 and 15 Celsius [38]. No adjustment was recommended for T ≥ 15, but for a daily average temperature of zero, corrected PM_10_ = 2.2 × TEOM. Changes were also suggested to TEOM operating procedures, including reducing the chamber temperature [9,59], reducing the flow rate to reduce filter clogging [38], and in Armidale, setting the many hours of negative TEOM PM_2.5_ pollution readings to zero [9].

Until 2009, the NSW EPA used the US EPA PM_10_ equivalence relationship for PM_2.5_:

corrected PM_2.5_ = 3 + 1.03 × TEOM

The 2008 NSW NEPM Compliance report [60] shows Liverpool’s annual average as 9.6 in 2008, compared to the 6.5 μg/m^3^ currently stored in the database and shown in Figure A3. These problems might help explain some of the discrepancies between the estimated 269 premature deaths from wood heater pollution in the study published in 2023 [20] and the 100 premature deaths in the study published in 2020 [51]. In 2010–2011, PM_2.5_ was measured by older-style TEOMs (before FDMS) with filter chambers heated to 50 C. This allowed the volatiles in the woodsmoke to evaporate, leading to the underestimation of woodsmoke PM_2.5_. As well as a slightly declining trend from 2006 to 2011, Figure A3 suggests a step increase when TEOMs were replaced with BAMs, starting with Liverpool, where the TEOM was replaced before winter (2 March 2012). Richmond’s TEOM was replaced on 7 September 2012, then Chullora (14 December 2012) and Earlwood (19 December 2012). The reduced loss of volatiles after BAMs were installed might explain why Liverpool’s annual average increased in 2011, compared to increases in 2012 for the other three sites. A comparison of the monthly average TEOM PM_2.5_ 2006–2011 vs. BAM PM_2.5_ (2013–18, Figure A4) shows discrepancies of up to 5 μg/m^3^ in winter.

In contrast to the multiple measurement techniques (DustTraks, TEOM, and HiVol samplers) in Tasmania, studies for Greater Sydney had to rely on models combining emissions inventory data with TEOM PM_2.5_ measured at a limited number of sites (four in Sydney). The 2010–2011 Greater Sydney study considered two different models [51]. Model CCAM-A attributed a much higher population-weighted PM_2.5_ exposure to wood heater pollution (1.39 μg/m^3^) than CCAM-B (0.49 μg/m^3^), but the former was noted to over-predict PM_2.5_ in winter by under-predicting the minimum temperatures and over-predicting light winds, so CCAM-B was considered marginally better [51]. However, after TEOMs were replaced with BAMs, the estimated contribution from wood heaters for 2013 in the study of Greater Sydney published in 2023 (1.261 μg/m^3^ PM_2.5_ [20]) is closer to the CCAM-A value of 1.39 than CCAM-B’s 0.49 μg/m^3^.
